# Particle-Bound Mercury Characterization in the *Central Italian Herbarium* of the Natural History Museum of the University of Florence (Italy)

**DOI:** 10.3390/toxics9060141

**Published:** 2021-06-15

**Authors:** Francesco Ciani, Laura Chiarantini, Pilario Costagliola, Valentina Rimondi

**Affiliations:** 1 Dipartimento di Scienze della Terra, Università di Firenze, Via G. La Pira 4, 50121 Firenze, Italy; francesco.ciani@unifi.it (F.C.); laura.chiarantini@unifi.it (L.C.); pilario.costagliola@unifi.it (P.C.); 2 Centro di Servizi di Microscopia Elettronica e Microanalisi (M.E.M.A.), Università di Firenze, Via G. Capponi, 50121 Firenze, Italy; 3 CNR-IGG, Via G. La Pira 4, 50121 Firenze, Italy

**Keywords:** mercury, trace elements, particulate matter, indoor air quality, pollution, museum

## Abstract

Museums air quality can be negatively affected by treatments with heavy metals compounds employed to prevent pest infestations. Among these, the past use of mercury dichloride (HgCl_2_) on herbaria artifacts currently produces high levels of indoor atmospheric gaseous mercury (Hg^0^) and possibly of particulate bound Hg (PBM), i.e., the particulate matter containing Hg. This study evaluates the PBM pollution in the *Central Italian Herbarium* (Natural History Museum of the University of Florence, Italy), characterizing the size range and chemical speciation with SEM-EDS microanalysis. The analysis of the total Hg concentration in the samples allowed to calculate the workers exposure risk to this pollutant. PBM is almost totally classifiable as fine particulate with a significant dimensional increase in a period of scarce attendance of the *Herbarium* rooms. The microanalysis indicates that Hg is essentially bound to S, highlighting the change of Hg speciation from the original association with Cl. The average Hg concentration reveals a potential health risk for workers as result of multiple Hg exposure pathways, mainly by ingestion. The study provides information for characterizing PBM pollution that could affect a workplace atmosphere and a useful basis to evaluate and correctly design solution strategies to reduce the contamination levels and protect workers’ health.

## 1. Introduction

The increasing attention devoted to the indoor air quality is the direct result of the high amount of time that people spend indoors during the day (about 88% for adults and 71–79% for children) in addition to the potential occurrence of airborne chemical and biological pollutants [1]. Indoor chemical contaminants are mainly due to anthropogenic sources, such as cooking/heating, tobacco smoking and the use of paints or cleaning products, while biological contaminants are mainly allergens, such as pollen grains and hair, or organisms, such as molds, mites or insects [2].

Indoor air quality is especially important in environments, such as museums, where the health of both visitors and workers must be protected. As evidenced by Schieweck [3], the specificity of the museum, i.e., the type of collections it contains and how they were treated to guarantee their protection, influence the air inside. This is, for example, the case of natural history collections where heavy metals (i.e., metals with atomic number greater than 20 and elemental density greater than 5 g cm^−3^, e.g., [4]) contamination is often observed as a direct consequence of pesticide treatments or taxidermal preparations [5,6]. This issue especially concerns mercury (Hg), which was highly employed in botanical collections, as shown in several studies carried out over the past few decades [6,7,8,9,10,11,12,13,14,15]. Mercury levels inside the atmosphere of herbaria are mainly due to the use of corrosive sublimate, an alcoholic solution of Hg dichloride (HgCl_2_), where plant samples were dipped to prevent cryptogamic or animal infestations until the middle of the last century [16]. The decomposition of HgCl_2_ causes the release of elemental gaseous Hg (GEM, Hg^0^) resulting in Hg^0^ levels ≥1000 ng m^−3^, i.e., the annual average reference value reported by World Health Organization [17] for inorganic Hg in the air, which can pose a health hazard for workers and museum visitors [15]. The reduction of Hg^2+^ to Hg^0^ has been attributed to several mechanisms: in the case of Hg in soils, Gustin [18] suggested a temperature-driven reduction, while Scholtz [19] assumed a photolytic reaction; in other studies carried out in herbaria, Oyarzun [9] hypothesized an enzymatic reduction, while Havermans [13] suggested a reduction reaction triggered by the cellulose of the paper on which plant samples are stored.

Mercury is ubiquitously distributed in the environment, naturally occurring in the Earth’s crust at average concentrations of about 0.05 mg kg^−1^ [20]. Mercury in the atmosphere is present both as gaseous forms, i.e., GEM and reactive gaseous Hg (RGM, Hg^2+^), and as particulate bound Hg (PBM). GEM represents the most abundant (>95%) form of Hg in the atmosphere, with high stability and long lifetime (from 0.5 to 2 years) thanks to chemical inertness [21]. Due to this long persistence, Hg is considered one of the major global environmental pollutants [22].

PBM consists of all airborne particulate containing Hg, including both stable condensed and gaseous forms adsorbed on atmospheric particulate matter (PM); it is operationally sampled and quantified by pulling air through a glass fiber or a quartz filter [23]. PBM usually includes all those particles with a diameter <2.5 µm, even if its characterization depends on the pore size of the filter used for its collection [24]. The accurate dimensional characterization is then essential to estimate the dry deposition of PBM, as well as any other particulate pollutant; the particles diameters directly influence gravitational sedimentation and the PBM residence time in the atmosphere [25]. In addition, PBM chemical speciation, as well as for the other Hg forms, is fundamental to understand PBM bioavailability and therefore the effects on human health [26].

**Figure 1:**
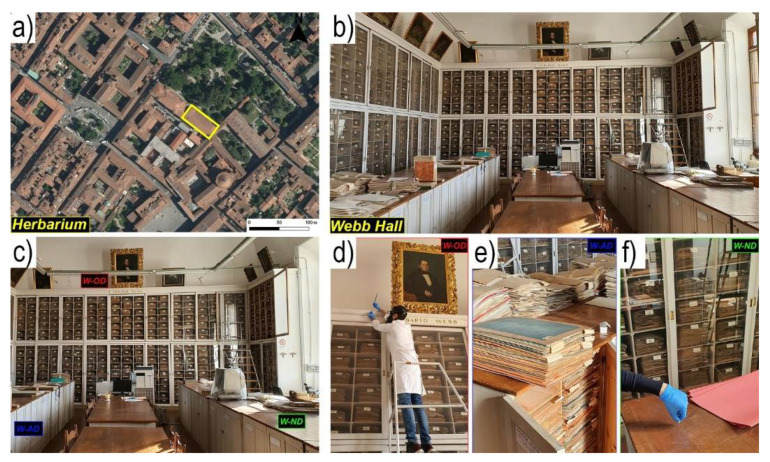
(**a**) Geographical location of the *Central Italian Herbarium* (Florence, Italy); (**b**) the *Webb Hall*; (**c**) detail location of the sampling points in the *Webb Hall* for the different dust types: (**d**) old-dust (W-OD), (**e**) almost-new dust (W-AD), and (**f**) new-dust (W-ND).

The study reported in this paper has been conducted in the *Central Italian Herbarium*, the botanical section of the Natural History Museum of the University of Florence (Italy), located in the historical center of the city (Figure 1a): with over 4.5 million plant samples, this herbarium is the largest in Italy and the tenth worldwide [27,28].

The aims of this paper are: (i) to evaluate the presence of Hg pollution in the PM of a working environment such as that of a botanical museum (ii) to characterize Hg particulate for size distribution and chemical composition; and (iii) to estimate the potential exposure risk for the museum’s workers exposed to PBM.

To our knowledge, this is the first attempt to characterize the morphometric and chemical speciation of Hg particulate in a botanical museum.

## 2. Materials and Methods

### 2.1. Study Site

The *Central Italian Herbarium* of Florence was founded in 1842 and hosts some historical collections, like the Herbarium Cesalpino (16th century), the Herbarium Micheli-Targioni (18th century), the Herbarium Webb (18th–19th century), and the Herbarium Beccari (19th century). HgCl_2_ was used as pesticide since the *Herbarium*’s foundation and despite this practice being abandoned more than a hundred years ago [29], recent studies still proved the persistence of high GEM concentrations (>50,000 ng m^−3^) [15]. The highest level of GEM was achieved in the so-called *Webb Hall* of the *Herbarium* (Figure 1b), hosting some of the most ancient and precious plant collections, such as the Webb and Beccari herbaria. 

### 2.2. PBM Ampling and Analysis

PBM sampling was carried out in October 2018 and in September 2020. In 2020, the sampling occurred soon after the lockdown period (March to May 2020) due to the COVID9 pandemic. Dust samples were collected in the *Webb Hall* (acronym for samples name W-) dabbing different areas (furniture, wall cornice, sample cabinet, study table) using a double-sided tape fixed on a stub. At each site, the same superficial area (ca. 1 × 1 m^2^) was sampled (three replicates for each sampling site) and the same number of dabbings (five) was done. Samples were roughly divided according to their deposition time (Figure 1c): (i) old dust (OD) from surfaces never dusted or hidden surfaces, like the frames of paintings hanging in the *Webb Hall* (Figure 1d); (ii) almost-new dust (AD), collected from surfaces cleaned approximately every two months, such as the cabinets that house plant samples (Figure 1e) or on book shelves; and iii) new dust (ND), recovered from surfaces cleaned about twice a week, such as desks and tables (Figure 1f). In addition, only during the 2020 sampling campaign, wooden pieces of a cabinet containing plant samples (inside which >50,000 ng m^−3^ of Hg^0^ were detected by Cabassi [15]) and paint fragments scraped from a wall were taken in the *Webb Hall*. As background sites, some dust samples (OD, AD, and ND) and wall paint fragments were also collected from rooms that host the Geomineralogy (acronym for samples name G-) and Botanical libraries (acronym for samples name B-) of the University of Florence, located on the ground level and first floor of the same building hosting the *Central Italian Herbarium*. It should be noted that the Botanical library hosted some Hg-poisoned plants collections in the first years following the *Herbarium*’s foundation (1842), and also in recent years for temporary exhibitions [27]. The complete list of samples with detailed descriptions and locations is shown in Table 1.

Dust samples were analyzed using scanning electron microscopy coupled to energy dispersive spectroscopy (SEM-EDS), using a model EVO-MA15 Zeiss, equipped with Oxford INCA 250 microanalysis software, located in the Interdepartmental Center for Electron Microscopy and Microanalysis Services (M.E.M.A.) of the University of Florence. All samples were metallized with a few nanometers of graphite and analyzed at a 15-kV acceleration voltage, 700 pA electron current, and at a 9–10 mm working distance. The analysis protocol was developed to standardize the measurements and quantify the particles composed of heavy metals in a faster and accurate way. Samples were previously investigated using an automatic procedure (Oxford INCA Feature automated analysis) that allowed to select the standard surface area of each sample to be investigated and a suitable magnification to identify the particles. Selecting backscattered electrons images with an appropriate black and white image threshold, the program is able to identify all particles comprising the selected thresholding levels, to morphologically measure them, to perform a rapid EDS (10-s of live-time) analysis, and thus chemically classify all dust particles. Heavy metal dust particles were recognized as light grey areas using backscattered electrons images with a contrast of 60%, brightness of 5–10%, and a threshold shade of grey (between 80 and 250) able to include all particles with an average atomic number (Z), heavier than Ba (reference standard BaSO_4_). In each stub, a total surface area of 4 mm^2^ was analyzed, observed in two distinct randomly selected 2 × 1 mm^2^ areas at 1000×. These methods allowed the isolation of dust particles characterized by a high average Z, comprising (but not exclusively) those containing Hg. It has to be stressed that, on the basis of backscattered electrons (BSE) thresholder images, the dimension (equivalent circular diameter, ECD) of the identified particles was smaller than the X-ray generation volume (i.e., the volume from which X-rays are produced). We could estimate an interaction volume with a diameter of about 0.8–1.1 μm (respectively at 10–20 kV acceleration voltages) for heavy elements/compounds (density > 8 g cm^−3^, i.e., Cu), and even greater (some microns) for light elements. To restrict SEM-EDS microanalyses to the volume of Hg particles, minimizing the contribution of the surrounding phases, a more detailed microanalysis (30-s of live-time) was selectively conducted on the stub surface areas where the largest Hg-particles (i.e., ECD > 1 μm or the largest for each sample) was found. All elements were re-calculated using the microanalysis software, which used the XPP matrix correction scheme developed by Pouchou and Pichoir [30]. This is a Phi-Rho-Z approach which uses exponentials to describe the shape of a φ(ρz) curve. The procedure is a “standard-less” semi-quantitative analysis employing pre-acquired standard materials. The monitoring of the analytical conditions (i.e., filament emission) was conducted with repeated analyses of a Co metallic standard.

Size differences among the Hg-particles were investigated using the non-parametric Mann–Whitney test, due to the non-normal distribution of data acquired in this study. The analyses were carried out using R-Studio software [31] with a significance level equal to 0.05 for all procedures.

### 2.3. Mercury Concentration and Health Risk Assessment

The total Hg concentration was determined from the dust samples collected from both the *Webb Hall* and the blank site (i.e., Geomineralogy library) using a direct Hg analyzer (Milestone DMA-80 evo, Department of Earth Sciences, University of Florence). The analysis was based on sample combustion, the pre-concentration of Hg on a gold amalgamator that was subsequently heated for Hg quantification with atomic absorption spectrometry (AAS). Dust (three replicates for each sampling site) for analysis was collected on the same double-sided tape used in the SEM-EDS analyses. To recover the sample weight, the tape was weighed before and after the dust collection using a 6 significant digits balance. A portion of a clean tape (blank) was also analyzed for Hg, and the value obtained subtracted from the dust samples. Analysis accuracy was verified using an international standard (BCR-280R, Hg = 1.46 ± 0.2 mg kg^−1^), and the error was within 10%.

To quantify the non-carcinogenic risk for workers from exposure to Hg-particles of the *Herbarium* dust, the average daily doses (ADD, mg kg^−1^ day^−1^) for the three pathways of exposure to the pollutant, i.e., non-dietary ingestion of particles (*ADD_ing_*), inhalation (*ADD_inh_*), and dermal absorption (*ADD_derm_*), were calculated with the following formulas, according to the Environmental Protection Agency of the United States (US EPA) Exposure Factors Handbook [32]:

*ADD_ing_* = (C_Hg_ × IR × EF × ED× RBA × CF)/(BW × AT) (1)

*ADD_inh_* = (C_Hg_ × IR × EF × ED)/(BW × AT × PEF) (2)

*ADD_derm_* = (C_Hg_ × SA × AF × ABS × EF × ED × CF)/(BW × AT) (3)

where C_Hg_ is the Hg concentration (mg kg^−1^) quantified in this study in the *Herbarium* dust; IR is the dust ingestion rate, considering two different scenarios with 30 mg day^−1^ (general population central tendency) and 60 mg day^−1^ (upper 90th percentile) due to the particular conditions of this working place, where a large amount of dust is produced by the degradation of organic materials (botanical samples and paper sheets) hosted in the halls; EF is the exposure frequency (days year^−1^) assumed to be 223 days in this study, corresponding to the average number of working days per year; ED is the exposure duration (years) established to 24 years; RBA is the relative bioavailability corresponding to 1 (unitless); CF is the unit conversion factor (10^−6^ kg mg^−1^); BW is the average body weight (70 kg); AT is the average time of exposure, calculated as ED × 365 days for non-carcinogenic substances; PEF is the particle emission factor (1.36× 10^9^ m^3^ kg^−1^); SA is the skin area (1070 cm^2^ for the hands only); AF is the skin adherence factor (0.07 mg cm^−2^); and ABS is the absorption factor for the skin (0.03, unitless). The coefficients IR, AT, PEF, AF and ABS were obtained from the US EPA [33,34,35,36]. A resume of the chosen parameters is reported in Table S1.

The hazard quotient (*HQ*), i.e., the potential for non-carcinogenic toxicity to occur, was then calculated for each *ADD* following the formulas below:

*HQ_ing_* = *ADD_ing_*/*RfD_ing_* (4)

*HQ_inh_* = *ADD_inh_*/*RfD_inh_* (5)

*HQ_derm_* = *ADD_derm_*/*RfD_derm_* (6)

where *RfD* is the specific reference dose, i.e., an estimation of the maximum permissible risk through a daily exposure [37]. The sum of the three *HQs* generates the hazard index (*HI*), i.e., the sum of multiple exposure pathways: if *HI* < 1 there is no risk for any pathogenic effects caused by Hg intake. Values of *HI* > 1 indicate a probability of non-carcinogenic effects, which increases as this value increases [32,33]; the toxic effects of Hg on human health involve neurological and behavioral disorders, systemic, reproductive and immunological toxicity, or adverse effects to the skin [37,38].

## 3. Results

### 3.1. Mercury Particles: Dimensions and Chemistry

**Figure 2:**
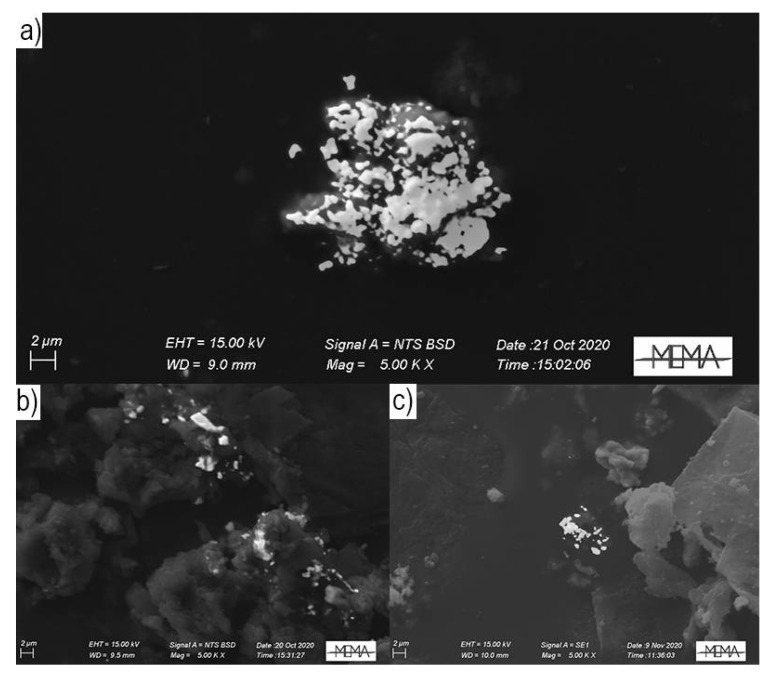
Mix of secondary electron (SE) and backscattered electron (BSE) images of the clusters of Hg-particles found in (**a**) W-OD, (**b**) W-AD and (**c**) W-ND, respectively.

**Figure 3:**
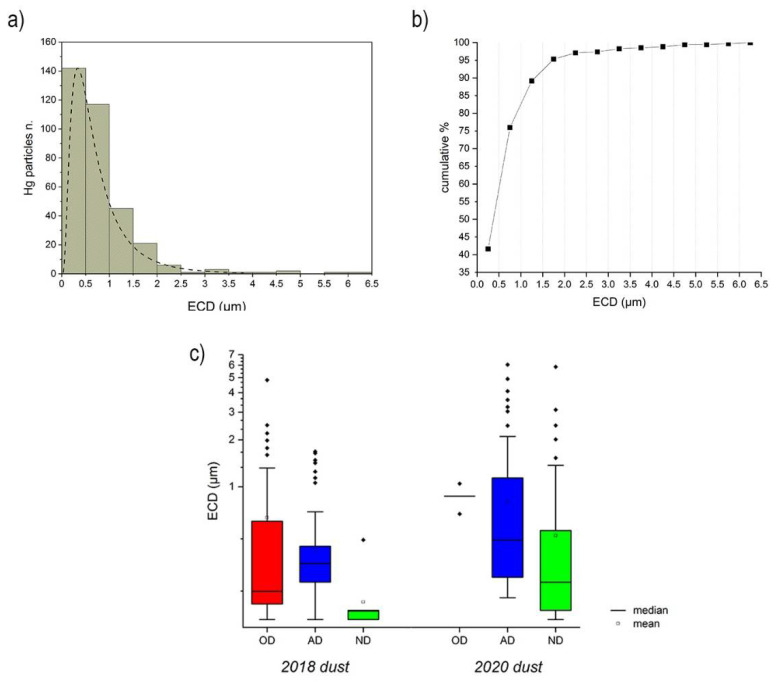
(**a**) Frequency distribution and (**b**) cumulative percentage of equivalent circular diameter (ECD) of all the Hg-particles in both the 2018 and 2020 sampling campaigns; (**c**) particle size ranges divided by year and dust type.

A total of 341 particles were observed and investigated for Hg in the *Herbarium* and background sites of this study using the 10-s live-time analyses. For the *Herbarium*, Hg-rich particles (*n* = 322) were observed in all samples (from old to new dust) collected from the *Webb Hall* in both the sampling campaigns. They generally occurred in clusters composed of several smaller grains (Figure 2) displaying a size distribution that followed a log-normal distribution (Figure 3a) with an average dimension of 0.79 μm. Rarely (<10%) Hg particles were observed to coalesce into larger grains, exceeding 1.5 µm in dimension (Figure 3b). The smaller dimensions were always recorded in the ND, with a constant dimensional sequence in the following order: ND < AD < OD. For both sampling campaigns, the highest number of Hg particles was observed in the W-AD (*n* = 62 in 2018, and *n* = 167 in 2020), while the lowest amounts were observed for the 2018 and W-ND (*n* = 6 and *n* = 2, respectively).

Significant differences were, however, observed between the 2018 and 2020 campaigns concerning dust average dimensions and abundances (Figure 3c and Table 2). In 2018, the Hg particles of the *Webb Hall* showed an average dimension of 0.67 µm. More in detail, the W-AD dust displayed an ECD ranging between 0.15 μm and 1.68 μm (average 0.59 μm). The larger size was instead reached by W-OD, with 23 Hg particles that showed an average ECD of 0.80 μm. The W-ND showed the lowest number (*n* = 6) and dimension (average ECD 0.26 μm) of Hg-particles (Figure 3c and Table 2).

In 2020 the *Webb Hall* Hg particles showed an average ECD of 0.85 µm (Figure 3 and Table 2), significantly higher than the Hg particles of the 2018 samples (Mann–Whitney test *p* < 0.05). Considering the specific dust categories, the size range for W-AD was between 0.29 and 5.90 μm (average ECD 0.90 µm), statistically higher (*p* < 0.05) than the W-AD of the 2018 samples. There were 55 Hg-bearing particles in the W-ND sample, with an average ECD of 0.57 µm and a maximum value of 6.25 μm; no statistical differences (*p* > 0.05) were observed comparing the W-ND samples from 2020 and 2018. The W-OD sample showed only 2 Hg particles with an average ECD of 0.95 μm. 

Regarding the dust samples collected at the background sites, only 18 Hg particles were detected in the Botanical library, considering both years of sampling; specifically, 12 in the B-AD sample from 2018 (average ECD 0.73 μm), 4 in the B-OD 2018 sample (average ECD of 1.11 μm), and 2 Hg particles in the B-OD 2020 sample. No Hg particles were found in B-ND (2018 and 2020), whereas in 2018, only one Hg particle was present in the G-ND sample of the Geomineralogy library (Table 2).

Similarly, no Hg particles were found in the other samples, such as wooden or paint fragments pieces collected in the *Webb Hall* (sample Webb-W) and in the libraries (samples W-P, B-P, and G-P).

**Figure 4:**
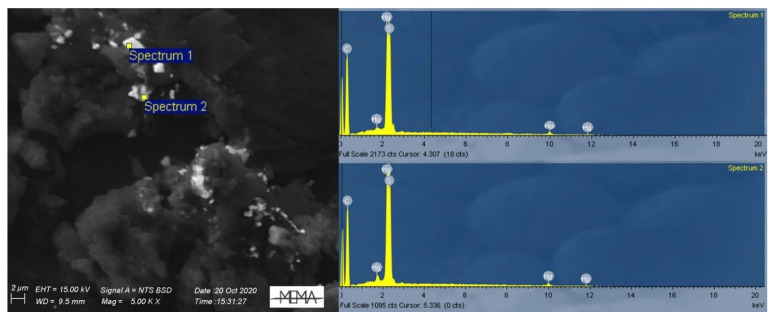
Mix SE and BSE image of the Hg-S compounds found in the 2020 W-AD, and the associated spectra of EDS analysis displaying Hg and S.

**Figure 5:**
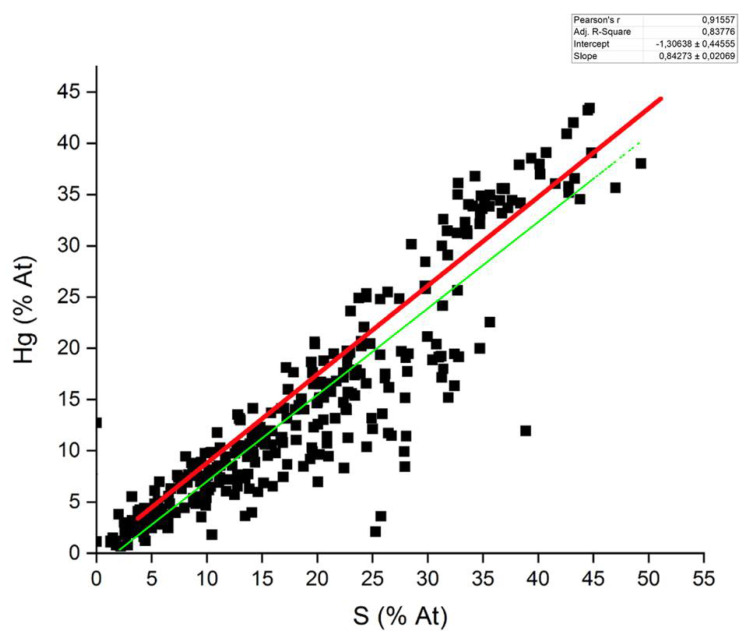
Atomic ratio (% At) between Hg and S in all the Hg particles found in both the sampling years in the *Webb Hall*. Green line and the coefficients refer to the regression analysis between the percentages of the two elements, while red line refer to the hypothetical 1:1 (atomic) ratio between these elements.

**Figure 6:**
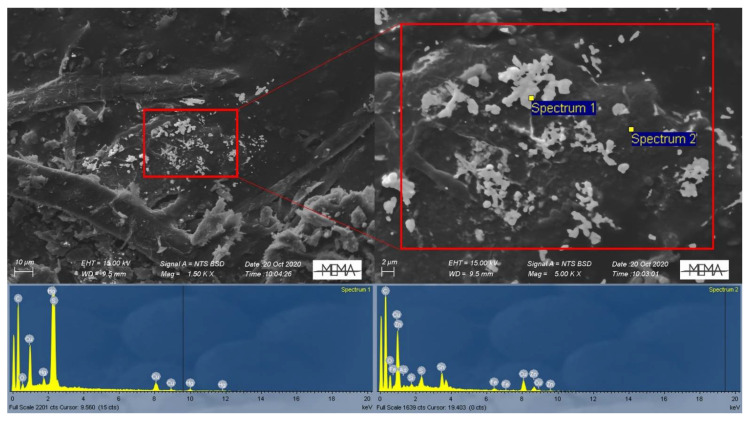
Detailed analysis of elemental composition of Hg particle clusters found in the 2020 W-AD (Spectrum 1) and of the area surrounding the Hg particles (Spectrum 2).

Interestingly, the results of the chemical characterization (30-s live-time SEM-EDS analysis) of the Hg particles (*n* = 54) only sporadically revealed the presence of Cl, the original anion to which Hg was bound when employed on plant samples. In only one particle of the 2020 W-OD sample Hg was indeed clearly associated with Cl, and hence ascribable to a Hg chloride. On the contrary, the element commonly found in the Hg particles was S (Figure 4), often displaying a Hg to S molar ratio of 1:1 (Figure 5). In these particles, O was notably absent. When O was present in low concentrations (~20% total weight), it probably came from the X-ray generation volume surrounding the Hg particles, as can be seen in Spectrum 2 of Figure 6. Similarly, we can explain the association of Cu to Hg in several sites of the 2020 W-AD sample as a volume halo effect (Figure 6).

The total Hg concentration (C_Hg_) found in the dust samples collected in the *Webb Hall* varied between 151 mg kg^−1^ (minimum) to 531 mg kg^−1^ (maximum), with an average value of 329 mg kg^−1^. The dust collected in the Geomineralogy library showed an average C_Hg_ of 13 mg kg^−1^ (min 4 mg kg^−1^, max 21 mg kg^−1^) (Supplementary File, Table S2).

### 3.2. Other Elements

In addition to Hg, the heavy metals found in other dust particles of the *Herbarium* and blank sites were mainly Zn, Ba, and Pb (Supplementary File, Table S3). In the 2018 sampling year, these elements were observed in greater amounts (*n* = 2075 in the *Webb Hall*, *n* = 3080 in the libraries) than in 2020 (*n* = 553 in the *Webb Hall*, *n* = 476 in the libraries); this decrease was especially marked for Pb (total *n* = 1532 in 2018 samples, total *n* = 143 in 2020 samples).

In the samples collected in the *Webb Hall*, the 2018 W-OD showed the highest number (*n* = 1098) of these three elements (Zn, Ba and Pb), while for the 2020 samples the highest number (*n* = 489) was reached by W-ND. The most represented heavy metal in both the sampling years was Ba.

In the 2018 samples, the dust particles containing heavy metals were more abundant in the libraries compared to the *Herbarium* halls: Zn particles were especially diffused in the Botanical library. The reverse was observed in 2020. The lowest number of heavy metal particles were always been found in the Geomineralogy library; during the 2020 campaign almost no heavy metal particles were found there.

The analysis carried on the wood pieces of a cabinet containing plant samples in the *Webb Hall* (sample W-W) revealed a very high level of Zn (946 particles) and Ba (930 particles), unlike the paint fragments (sample W-P) that showed only a few particles (*n* = 8) containing heavy metals.

## 4. Discussion

### 4.1. PBM Pollution in the Central Italian Herbarium

The Hg particles of the *Central Italian Herbarium* are classifiable as fine particulate (i.e., PM2.5), generally showing (>90%) an ECD < 1.5 µm (Figure 3b). Based on the European standards for workplace atmospheres [39], this fraction must be included in the respirable one, i.e., the PM that can reach the unciliated airways tract of human lungs. 

The most relevant exposure routes for PBM are inhalation and non-dietary ingestion, as well as dermal absorption by the skin [40]. The inhaled fraction is especially harmful to human health, as it can penetrate almost completely into the deepest parts of the respiratory tract, at the gas-exchange region [41]. These dangerous effects are amplified due to the possible involuntary ingestion of inorganic/organic Hg particles that are, respectively, partially/totally absorbed in the gastrointestinal tract [22,42]. In this regard, the analysis for health risk assessment for the dust samples collected in the *Webb Hall* (Supplementary File, Table S2) considering a 30 mg day^−1^ normal dust ingestion rate (the general population central tendency according to the US EPA [35]) revealed a cumulative hazard risk (*HI)* ranging between 0.3 (min) and 1.2 (max), depending on the different concentration of Hg in the dust (151–531 mg kg^−1^), with an average value of 0.7 that indicates no potential adverse effects (*HI* < 1). Nevertheless, we also depicted a scenario considering an ingestion rate of 60 mg day^−1^ (the upper 90th percentile for general population), due to the special conditions of *Herbarium* environment, where organic samples (paper and exsiccated plants) are stored for long time and pulverized without daily cleaning practices. In this case, the *HI* values were >1 (*HI* = 1.2–2) considering the average and maximum C_Hg_, hence indicating a significant potential risk from multipath Hg exposures for *Herbarium* workers. In the risk calculation (i.e., *HI*), dust ingestion contributes the most (*HQ_ing_* = 0.9–1.5), clearly indicating involuntary ingestion as the principal exposure route to be controlled for in terms of health safety in the *Herbarium* museum. The *HI* and *HQ* values do not significantly vary, even considering specific exposures for women or men (data for women, BW = 70 kg; SA = 890 cm^2^, not shown). It should be also noted that a relative bioavailability (RBA) = 1 was considered for dust ingestion, as recommended by the US EPA [43]. However, bioavailability varies considerably among Hg species [26] (see below), and therefore we must consider this as a precautionary measure, as the worst scenario for the protection of worker health.

On the contrary, no potential health risk (*HI* < 1) was evidenced based on the analyses of the dust samples collected from the background site, i.e., the Geomineralogy library (Supplementary File, Table S2). Even if the Hg concentration obtained here does not indicate a health risk, Hg contents are especially high when compared to those of the mean crustal values (~50 µg kg^−1^), which is not surprisingly considering that indoor environments generally exceed natural concentrations or even street dust [44]. These Hg levels are in the upper range of those observed in house dust sampled in several residences in Ottawa (Canada) [44] or in other Chinese cities [45]. 

The highest amount of Hg particles was observed in the *Herbarium* hall where the most ancient plant collections of the *Herbarium* are conserved. Concordantly, as much as 531 mg kg^−1^ Hg was observed in the room dust investigated in this study, comparable or higher than the levels observed in other museums holding natural collections [6,46]. This is especially evident on the basis of the results of the libraries samplings, where almost no Hg particles were found, with the exception of the botanical one, where some herbarium samples were stored in the past. As evidenced in other studies [47,48,49], the main sources of pollutants in PM often come from indoor contamination; therefore, an external origin must be excluded.

Data of this study clearly evidences PBM characterization being of fundamental importance for human health, especially in those contexts like the *Central Italian Herbarium* where gaseous forms are not the only airborne Hg species. In addition, it should be noted that the legislative workplace exposure limits in Italy refer to all inorganic Hg compounds; PBM is, therefore, included in this definition and should be quantified [50].

The highest number of Hg particles has always been found in the AD, i.e., the dust collected inside cabinets where plant samples are stored and where GEM concentrations reach the highest values (>50,000 ng m^−3^) [15]; this evidence underlines that the HgCl_2_-treated plant collections are clearly the main pollution source and therefore *Herbarium* workers are particularly exposed to Hg when handling old samples. 

It is worth noting that the number and the dimension (i.e., ECD) of particles increased over time. As evidenced by Xiu [51], the PBM formation mechanism may be due to direct emissions (from anthropogenic or natural sources) or it could be the result of adsorption of Hg gaseous fraction on PM and its chemical transformation. Successive changes in PBM size may be due to chemical or physical process, such as nucleation and adsorption, and environmental conditions, such as temperature and humidity [25]. In periods of scarce cleaning of surfaces, such as those following the COVID-19 pandemic lockdown in our study, Hg particles tended to coalesce (Figure 2). Concordantly, the newly formed dust almost always had a significantly lower dimension (Mann–Whitney test, *p* < 0.05) than the OD and the AD of the respective years.

On the other hand, the pandemic period contributed to a drastic decrease in PBM as well as the other heavy metals in the samples taken in libraries, which is likely linked to the radical cleaning to which these rooms were subjected to re-opening to the public, especially to students.

### 4.2. PBM Composition and Other Elements

Mercury treatment of herbaria collections occurred in the form of corrosive sublimate (HgCl_2_), hence a Hg chloride compound. After application on a plant specimen, this compound probably firstly reduces to calomel (Hg_2_Cl_2_) due to its reaction with the plant matrix or water [52], and then to gaseous Hg^0^ via reaction with the cellulose carbonyl group of the paper supporting the plant samples [13]. This last Hg_2_Cl_2_ to Hg^0^ step was supported by the study of Passerini and Pampanini [29]; the analysis carried out by these authors revealed the presence of only Hg^0^ blackening the paper sheets where some plants of the *Central Italian Herbarium* are mounted, while, on the plant samples, calomel and organic Hg compounds were found. The unique Cl-rich particle of our study, with a 1:1 molar ratio, confirms the likely intermediate conversion of HgCl_2_ to calomel before reduction to Hg^0^. Except this particle, Cl is systematically absent from the investigated dust. As detailed by the microanalyses, the prevalent anion associated with Hg in the *Herbarium* PM was S, often in a molar ratio of approximately 1:1 with Hg (Figure 5), suggesting the presence of a Hg sulfide and the reaction of Hg with S over time. The direct association between Hg and S is also evidenced by the Figure 6 spectra, which confirms the absence of S from the X-ray generation volume surrounding the Hg particles. The solubility and bioavailability of the Hg sulfide compounds were very low compared to the other Hg compounds; absorption of cinnabar (HgS) by the gastrointestinal tract is very scarce (0.05%) compared to HgCl_2_ (7–15%) and to organo-Hg compounds (almost totally absorbed) [53]. Based on these different bioavailabilities, we also depicted the risk scenario setting a RBA = 0.05, and obtaining *HI* indexes that were always <1, independently of the ingestion rates (Table S2). Full understanding of Hg speciation in the *Herbarium* dust is therefore of utmost importance. 

The constant presence of S bound to the Hg particles had two possible explanations. First, we cannot rule out that S came from the treatments to disinfect the plants before their acquisition by the museum; Signorini [16] reported the widespread use of fumigation with CS_2_ on plant samples, a pest control practice carried out until the early 1970s, and also in the *Central Italian Herbarium* (personal communication Dr. Piero Cuccuini, former Curator of the Botanical Section of the Natural History Museum of Florence). On the other hand, based on the microanalyses conducted on the wooden fragments of a cabinet of the *Webb Hall* (sample Webb-W, Table S3), another S source could be represented by BaSO_4_. This compound was widely used since the 19th century as an inorganic synthetic pigment for white paint, particularly for lead white, but it was also employed for artistic purposes either in the form of *blanc fixe* (pure BaSO_4_) or as lithopone (BaSO_4_ + ZnS), obtained by precipitating BaSO_4_ with BaS [54,55]. Lithopone may indeed also explain the simultaneous massive presence of Zn and Ba in the wooden fragments of the *Webb Hall* (Table S3); the cabinets hosting plant samples were white painted and date back to the original period of the *Herbarium*’s foundation (Figure 1b).

Similarly, we can explain the widespread presence of Cu in the 2020 W-AD samples (Figure 6) as a derivation from the blue–green pigment associated with the packages of cardboard containing the samples (as can be seen in Figure 1e). This is probably verdigris, a mixture of copper acetate, carbonate, and chloride, which was widely used in manuscripts and early printed books until the introduction of synthetic pigments [56]. It has to be stressed that Cu is probably not directly associated with Hg particles, but it originates from the X-ray generation volume that surrounds them (Figure 6).

Finally, the drastic reduction of Pb-particles in the 2020 samples, both from the *Herbarium* and from libraries has to be underlined (Table S3). Unlike the other elements, for which an internal source of contamination is easily presumed, for Pb, the contamination could come from outdoor environments [57]. From this point of view, it should be noted that *Herbarium* rooms are not hermetically closed, and there is a ventilation and air recirculation system that could be the cause of contamination from the outside. However, despite a general decrease of Pb being observed in the outdoors during the pandemic period [58,59], in our case we can probably exclude this origin. One of the major causes of indoor Pb pollution, excluding cooking and tobacco smoking, is the use of old Pb-based paints [60], but in our case, the microanalyses of the fragments scrapped from the wall (sample W-P) and from the cabinet wood of the *Webb Hall* (sample W-W) do not support this hypothesis. The causes of Pb reduction are not entirely clear; perhaps, Pb-sources (probably rooms painted with Pb-based paints) are present in the building hosting both the *Herbarium* and the libraries and, differently from PBM—the scarce attendance during the first months of 2020 may have influenced the decrease of the indoor Pb levels.

## 5. Conclusions

The results of the present study clearly indicate that PBM constitutes a non-negligible source of Hg pollution that affects the *Central Italian Herbarium* atmosphere; preliminary information about PBM dimensional and elemental characteristics have been collected.

The PBM size range is almost totally (>95%) classifiable as fine particulate (≤2.5 µm), particularly harmful to human health, although a dimensional increase likely associates with time due to the coalescence phenomena. As well as the size range, the chemical composition also changed over time, underlying that HgCl_2_ solution used to treat plant samples still sublimates. Sulfur availability in the environment, possibly due to fumigation treatments of plant specimens with CS_2_ and/or the employment of BaSO_4_ as white pigment in the *Webb Hall* favors Hg recombination with S and the precipitation of Hg sulfide, which is, at present, the main Hg species found in PBM.

In the worst-case scenario (100% bioavailability of Hg), the risk analysis for PBM indicates a multiway exposure risk for human health; among these, the main dangerous pathway is ingestion, suggesting that *Herbarium* workers should avoid any activity that promotes involuntary dust intake (for example drinking or eating in the Museum halls), as well as wearing safety equipment (such as gloves and lab coats) when attending these rooms. Fortunately, data of this study suggest a lower bioavailability of Hg species in *Herbarium* dust (i.e., Hg sulfides), which should consistently lower the health risk for workers in the Museum. Further studies to fully elucidate the Hg mineral phases in the *Herbarium* dust are planned for the near future. 

This research provides information on an abatement system that could be installed to reduce the Hg pollution (i.e., gaseous and particulate fraction) that affects the *Herbarium*. This would need to be supplied with filters able to retain fine PM. In addition, the proper management of plant samples could be of fundamental importance to reduce the health risks for both *Herbarium* workers and visitors.

## Figures and Tables

**Table 1:** Location and description of the sampling sites.

Sampling Site	Sample Name	Dust Type	Location
*Webb Hall*	W-OD	old	Above the top shelf of the closet surrounding the Webb Hall
	W-AD	almost-new	On the surface of a sample pack inside a cabinet of the Webb Hall
	W-ND	new	On the shelf of a cabinet hosting herbaria samples of the Webb Hall
	W-W	wood	Wooden pieces of a cabinet of the Webb Hall
	W-P	paint	Paint fragments scraped off the wall of the Webb Hall
Botanical library	B-OD	old	On the upper frame of a cabinet in the Botanical library
	B-AD	almost-new	Inside a closet of the Botanical library
	B-ND	new	On the support surface of a cabinet in the Botanical library
	B-P	paint	Paint fragments scraped off the wall of the Botanical library
Geomineralogy library	G-OD	old	On the upper frame of a cabinet in the Geomineralogy library
	G-AD	almost-new	On a book shelf on the upper balcony in the Geomineralogy library
	G-ND	new	On a study table in the Geomineralogy library
	G-P	paint	Paint fragments scraped off the wall of the Geomineralogy library

**Table 2:** Results of the dimensional analysis of particulate bound mercury (PBM); for samples names, refer to Table 1.

	2018					2020				
Sample	ECD (μm)					ECD (μm)				
	Hg-Particles n.	Min	Max	Average	SD	Hg-Particles n.	Min	Max	Average	SD
W-OD	30	0.15	4.82	0.80	1.01	2	0.83	1.10	0.95	-
W-AD	62	0.15	1.68	0.59	0.34	167	0.29	5.90	0.90	0.80
W-ND	6	0.15	0.66	0.26	0.20	55	0.20	6.25	0.57	0.93
W-W	n.a.	n.a.	n.a.	n.a.	-	-	-	-	-	-
W-P	n.a.	n.a.	n.a.	n.a.	-	-	-	-	-	-
B-OD	4	0.83	1.91	1.11	0.52	2	0.51	2.78	1.65	-
B-AD	12	0.42	1.44	0.73	0.36	-	-	-	-	-
B-ND	-	-	-	-	-	-	-	-	-	-
B-P	n.a.	n.a.	n.a.	n.a.	-	-	-	-	-	-
G-OD	-	-	-	-	-	-	-	-	-	-
G-AD	-	-	-	-	-	-	-	-	-	-
G-ND	1	0.51	0.51	0.51	-	-	-	-	-	-
G-P	n.a.	n.a.	n.a.	n.a.	-	-	-	-	-	-

## Data Availability

All supporting data have been included in this study, and are available from the corresponding authors upon request.

## Supplementary Materials

The following are available online at https://www.mdpi.com/article/10.3390/toxics9060141/s1, Table S1: Summary of the parameters employed for dust ingestion (*ADD_ing_*), inhalation (*ADD_inh_*), and dermal absorption (*ADD_derm_*) calculation, Table S2: Results of the hazard quotients (*HQ*) for the three different exposure pathways (ingestion, inhalation and dermal absorption) and the calculated hazard index (*HI*) for the dust samples collected in the *Central Italian Herbarium* and in the Geomineralogy library (the background site), Table S3: Results of the dimensional analysis of the other heavy metals found in the samples dust of both years.
