# Supplementary material for: Particle-Bound Mercury Characterization in the Central Italian Herbarium of the Natural History Museum of the University of Florence (Italy)
Source: Toxics. 2021 Jun 15;9(6):141. doi: 10.3390/toxics9060141 (PMC8232298; doi:10.3390/toxics9060141)
Supplement: Supplementary file 1 [file toxics-09-00141-s001.zip › toxics-1232586-supplementary.pdf]

toxics

MDPI

Article

Supplementary Materials: Particle-Bound Mercury Characterization in the Central Italian Herbarium of the Natural History Museum of the University of Florence (Italy)

Francesco Ciani, Laura Chiarantini, Pilario Costagliola and Valentina Rimondi

Table S1. Summary of the parameters employed for dust ingestion (ADD_{ing}), inhalation (ADD_{inh}), and dermal absorption (ADD_{derm}) calculation.

Parameter	Value	Reference
C_{Hg} (Hg concentration – mg kg^{-1})	4 - 691	This study
IR (ingestion rate – mg day^{-1})	30 - 60	US EPA (2017)
EF (exposure frequency – days year^{-1})	223	This study
ED (exposure duration - years)	24	US EPA (2004)
RBA (relative bioavailability)	1	US EPA (2007)
	0.05	Welfringer and Zagury (2009)
CF (conversion factor – kg mg^{-1})	1E-06	US EPA (2002)
AT (average time)	8760	US EPA (2002)
BW (average body weight)	80	US EPA (2011)
InhR (inhalation rate - m^3 day^{-1})	15.85	US EPA (2017)
PEF (particle emission factor - m^3 kg^{-1})	1.36E+09	US EPA (2002)
SA (skin area- cm^2)*	1070	US EPA (2011)
AF (skin adherence factor – mg cm^{-2})	7.0E-02	US EPA (2002)
ABSd (dermal absorption factor - mg cm^{-2})	3.0E-02	US EPA (2004)
Rfd_ing (reference dose for ingestion)	1.6E-04	RAIS (2020)
Rfd_inh (reference dose for inhalation)	3.0E-04	RAIS (2020)
Rfd_derm (reference dose for dermal absorption)	2.1E-05	RAIS (2020)

* hands only.

Table S2. Results of the hazard quotients (*HQ*) for the three different exposure pathways (ingestion, inhalation and dermal absorption) and the calculated hazard index (*HI*) for the dust samples collected in the *Central Italian Herbarium* and in the Geomineralogy library (the background site) of the University of Florence. Four different scenarios have been outlined, based on the ingestion rate (IR) suggested by US EPA (general population central tendency and 90th upper percentile) and the relative bioavailability (total = 1 or partial = 0.05, based on Welfringer, B. and Zagury, G. J., 2009) of the Hg compounds.

		**Central Italian Herbarium**			**Geomineralogy Library**		
		**min [Hg] (151 mg kg $^{-1}$)**	**max [Hg] (531 mg kg $^{-1}$)** *(St. Err. 65 mg/Kg)*	**average [Hg] (329 mg kg $^{-1}$)**	**min [Hg] (4 mg kg $^{-1}$)**	**max [Hg] (21 mg kg $^{-1}$)** *(St. Err. 6 mg/Kg)*	**average [Hg] (13 mg kg $^{-1}$)**
RBA = 1		*IR= 30 mg day $^{-1}$ (general population central tendency)*					
	HQ_{ing}	0.2	0.8	0.4	< 0.1	< 0.1	< 0.1
	HQ_{inh}	< 0.1	< 0.1	< 0.1	< 0.1	< 0.1	< 0.1
	HQ_{derm}	0.1	0.4	0.3	< 0.1	< 0.1	< 0.1
	HI	0.3	1.2	0.7	< 0.1	< 0.1	< 0.1
		IR= 60 mg day $^{-1}$ (general population 90th upper percentile)					
	HQ_{ing}	0.4	1.5	0.9	< 0.1	0.1	< 0.1
	HQ_{inh}	< 0.1	< 0.1	< 0.1	< 0.1	< 0.1	< 0.1
	HQ_{derm}	0.1	0.4	0.3	< 0.1	< 0.1	< 0.1
	HI	0.6	2.0	1.2	< 0.1	0.1	< 0.1
RBA = 0.05		*IR= 30 mg day $^{-1}$ (general population central tendency)*					
	HQ_{ing}	< 0.1	< 0.1	< 0.1	< 0.1	< 0.1	< 0.1
	HQ_{inh}	< 0.1	< 0.1	< 0.1	< 0.1	< 0.1	< 0.1
	HQ_{derm}	0.1	0.4	0.3	< 0.1	< 0.1	< 0.1
	HI	0.1	0.5	0.3	< 0.1	< 0.1	< 0.1
		IR= 60 mg day $^{-1}$ (general population 90th upper percentile)					
	HQ_{ing}	< 0.1	0.1	< 0.1	< 0.1	< 0.1	< 0.1
	HQ_{inh}	< 0.1	< 0.1	< 0.1	< 0.1	< 0.1	< 0.1
	HQ_{derm}	0.1	0.4	0.3	< 0.1	< 0.1	< 0.1
	HI	0.1	0.5	0.3	< 0.1	< 0.1	< 0.1

Table S3. Results of the dimensional analysis of the other heavy metals found in the samples dust of both years.

**2018 dust**	**Zn**	**ECD (μm)**			**Ba**	**ECD (μm)**			**Pb**	**ECD (μm)**		
	**Particles n.**	**min**	**max**	**average**	**Particles n.**	**min**	**max**	**average**	**Particles n.**	**min**	**max**	**average**
W-OD	251	0.15	11.96	0.79	605	0.15	11.96	0.86	242	0.15	3.64	0.74
W-AD	69	0.15	4.20	0.69	315	0.15	8.14	0.68	318	0.15	10.8	0.63
W-ND	64	0.15	1.61	0.57	185	0.15	10.04	0.80	26	0.15	1.91	0.57
B-OD	272	0.29	2.63	0.72	192	0.29	2.62	0.79	93	0.29	2.62	0.81
B-AD	439	0.29	2.55	0.85	550	0.29	2.65	0.86	788	0.29	2.50	0.78
B-ND	248	0.29	2.60	0.82	121	0.29	2.56	0.79	20	0.29	2.1	0.92

G-OD	61	0.29	1.97	0.89	66	0.29	2.35	0.85	17	0.29	2.35	0.8
G-AD	129	0.29	2.65	0.8	58	0.29	2.58	0.90	26	0.29	1.9	0.8
G-ND	4	0.42	1.47	0.78	9	0.51	2.63	1.11	1	1.02	1.02	1.02
W-W	n.a.	n.a.	n.a.	n.a.	n.a.	n.a.	n.a.	n.a.	n.a.	n.a.	n.a.	n.a.
W-P	n.a.	n.a.	n.a.	n.a.	n.a.	n.a.	n.a.	n.a.	n.a.	n.a.	n.a.	n.a.
B-P	n.a.	n.a.	n.a.	n.a.	n.a.	n.a.	n.a.	n.a.	n.a.	n.a.	n.a.	n.a.
G-P	n.a.	n.a.	n.a.	n.a.	n.a.	n.a.	n.a.	n.a.	n.a.	n.a.	n.a.	n.a.

**2020 Dust**	**Zn**	**ECD (μm)**			**Ba**	**ECD (μm)**			**Pb**	**ECD (μm)**		
	**Particles n.**	**min**	**max**	**average**	**Particles n.**	**min**	**max**	**average**	**Particles n.**	**min**	**max**	**average**
W-OD	3	0.29	0.51	0.40	3	0.29	0.51	0.40	10	0.29	1.25	0.78
W-AD	37	0.29	1.97	0.69	-	-	-	-	11	0.29	1.66	0.74
W-ND	205	0.15	1.70	0.40	280	0.15	2.43	0.44	4	0.15	0.47	0.25
B-OD	153	0.29	4.31	0.84	158	0.29	4.32	0.91	31	0.42	5.66	1.36
B-AD	15	0.29	1.74	0.74	31	0.29	1.93	0.61	79	0.29	1.28	0.62
B-ND	2	0.41	0.65	0.53	4	0.41	1.25	0.83	-	-	-	-
G-OD	-	-	-	-	2	0.29	4.25	2.28	-	-	-	-
G-AD	-	-	-	-	1	0.51	0.51	0.51	-	-	-	-
G-ND	-	-	-	-	-	-	-	-	-	-	-	-
W-W	946	0.06	4.10	0.45	930	0.06	4.10	0.45	8	0.19	0.97	0.50
W-P	2	0.97	1.01	0.99	6	0.72	1.28	0.93	-	-	-	-
B-P	-	-	-	-	-	-	-	-	-	-	-	-
G-P	-	-	-	-	1	0.14	0.42	0.28	-	-	-	-
